# AKT regulation of mesothelial-to-mesenchymal transition in peritoneal dialysis is modulated by smurf2 and deubiquitinating enzyme USP4

**DOI:** 10.1186/s12860-015-0055-7

**Published:** 2015-03-06

**Authors:** Li Xiao, Xiang Peng, Fuyou Liu, Chengyuan Tang, Chun Hu, Xiaoxuan Xu, Ming Wang, Ying Luo, Shikun Yang, Panai Song, Ping Xiao, Yashpal S Kanwar, Lin Sun

**Affiliations:** Department of Nephrology, Second Xiangya Hospital, Central South University, Changsha, Hunan 410011 China; Departments of Pathology & Medicine, Northwestern University, Chicago, USA

**Keywords:** Peritoneal dialysis, Akt, Smurf2, Smad7, USP4

## Abstract

**Background:**

Transforming growth factor-β1 (TGF-β1) plays a key role in mesothelial-to-mesenchymal transition (MMT) during peritoneal dialysis (PD). However, the role of Akt in MMT transformation in PD is not clear.

**Results:**

In this study, we observed that the phosphorylated form of protein kinase B (Akt), termed as pAkt, was up-regulated in the peritoneum of mice undergoing PD. It was associated with thickening of the peritoneum and up-regulation of TGF-β1. Upregulation of pAkt paralleled with the increased expression of Smad ubiquitination regulatory factor 2 (Smurf2), Vimentin and fibronectin (FN), and decreased expression of mothers against decapentaplegic homolog 7 (Smad7) and Zonula Occludens protein 1(ZO-1) in mice undergoing PD treatment and in TGF-β1 induced human peritoneal mesothelial cells (HPMCs). These changes were reversed with the treatment of a PI3K/Akt inhibitor LY294002 *in vivo* or in cells transfected with Akt dominant-negative (Akt-DN) plasmids *in vitro*. Increased Smurf2 expression in HPMCs, induced by TGF-β1 was accompanied with altered expression of Transforming growth factor receptor I (TβR-I), Smad7, ZO-1, Vimentin and FN *via* Akt modulation. In addition, inhibition of Ubiquitin carboxyl-terminal hydrolase 4 (USP4) decreased TGF- β1-induced expression of TβR-I and reversed the altered expression of Smad7, Smurf2, ZO-1 and Vimentin. Moreover, TGF-β1 accentuated the interactions between Smurf2 and Smad7, while reduced the association between TβR-I and Smurf2. These interactions were reversed by the treatment of Akt-DN and USP4 siRNA, respectively.

**Conclusions:**

These data implied that Akt mediated MMT in PD *via* Smurf2 modulation/and or Smad7 degradation while conceivably maintaining the TβRI stability, most likely by the USP4.

## Background

Continuous ambulatory peritoneal dialysis (CAPD) is an alternative therapy to hemodialysis for the treatment of end-stage renal disease. During CAPD, the peritoneum is exposed to bio-incompatible dialysis fluids that cause peritoneal fibrosis (PF) by denudation of mesothelial cells. Thus, PF is a common morphological change in peritoneal dialysis (PD) patients and in PD animal models [1]. This change is associated with impaired peritoneal membrane function, ultrafiltration failure culminating into discontinuation of dialysis [2]. Furthermore, epithelial-mesenchymal transition (EMT) of peritoneal mesothelial cells (termed as MMT) plays a crucial initiating role in the progression of PF [3]. Many factors, including high glucose (HG), hypertonicity, low pH, glucose degradation products and advanced glycation end-products (AGEs) can induce Transforming growth factor-β1 (TGF-β1) activation, and blocking of TGF-β1 signaling protects the peritoneal membrane from dialysate-induced damage [4,5]. Therefore, TGF-β1 has been regarded as the primary regulator and plays a central role in MMT and fibrosis of peritoneal tissue during PD.

Recent studies suggest that phosphatidylinositol 3-kinase kinase (PI3K) /AKT signaling not only plays an important role in the regulation of cell survival, growth, metabolism and migration [6], but also modulates EMT transformation that may be independent of TGF-β1/Smad signaling [7]. In addition, PI(3)K/AKT activation induced by TGF-β through the direct binding of Type I transforming growth factor receptor (TβRI) to PI(3)K [8] is related to increased ECM protein expression [9]. Furthermore, TGF-β1 induces fibrosis in various cell lines including human lung cancer cells, hepatocellular carcinoma and renal proximal tubule cells *via* the PI3K/Akt signaling pathways [10-12]. On the other hand, it has been reported that Akt modulates E3 ubiquitin ligase, such as the transcription of Smad7 ubiquitination regulatory factor2 (Smurf2) that is induced by TGF-β1 [13], indicating that TGF-β1/Akt/smurf2 pathway may play a critical role in some pathophysiological conditions. Furthermore, it has been reported that Smurf2 contributes to a reduction of Smad7 in fibrosing UUO kidneys [14]. The Smurf2 levels have been reported to be increased in early period of fibrosis in rat liver and TGF-β1-treated LX-2 cells, and they are accompanied with reduced levels of Smad7 [15]. Thus, it seems that Smad7 provides a negative feedback to TβR1 by binding to Smurf2 and brings Smurf2 to the activated TβR for their polyubiquitination and degradation [16]. This would indicate that decreased levels of Smad7 may lead to activation of TGF-β1 signaling. It has been demonstrated that Smad7 expression is decreased in peritoneum of PD patients. Overexpression of Smad7 inhibits Smad2/3 activation and the EMT related protein expression, extracellular matrix protein (ECM) and fibrosis in the peritoneal mesothelial cells and animal models of PD [17-19]. Whether Akt induces Smurf2 expression and then inhibits Smad7 participation in MMT transformation during PD and the relevant mechanism(s) involved have not been thoroughly explored.

The TGF-β receptors (TβR) play a key role in TGF-β signaling pathway, which is targeted for ubiquitylation-mediated degradation by the Smad7/Smurf2 complex [20]. Emerging studies have demonstrated that deubiquitinating enzymes (DUBs) play a key role for maintaining TβRI stability. Among of them, ubiquitin-specific peptidase-4 (USP4) and-15 (USP15) extend the life of activated TβRI and are against the negative pressure of TβRI-ubiquitinating complexes [16,21]. Interestingly, it was also found that Akt directly associates and phosphorylates USP4, and then induces the translocation of USP4 from the nucleus to the cytoplasm and plasmalemma for maintaining TβRI stability [22]. Therefore, USP4 mediates TβRI regulation *via* PI3K/Akt pathway, which is a strong modulator of TGF-β pathway and plays a critical role in the crosstalk between TGF-β and AKT signaling. Whether Akt mediates MMT transformation in PD fibrosis and the mechanism(s) by which USP4 is involved in this process has yet to be elucidated.

In the present study, we investigated that if increased activation of Akt exerts a critical effect on TGF-β1 induced MMT in PD *via* Smurf2/Smad7 complex and USP4/TβRI pathway.

## Results

### Expression of TGF-β1 and p-Akt, Smurf2 and Smad7 in PD mice

**Figure 1:**
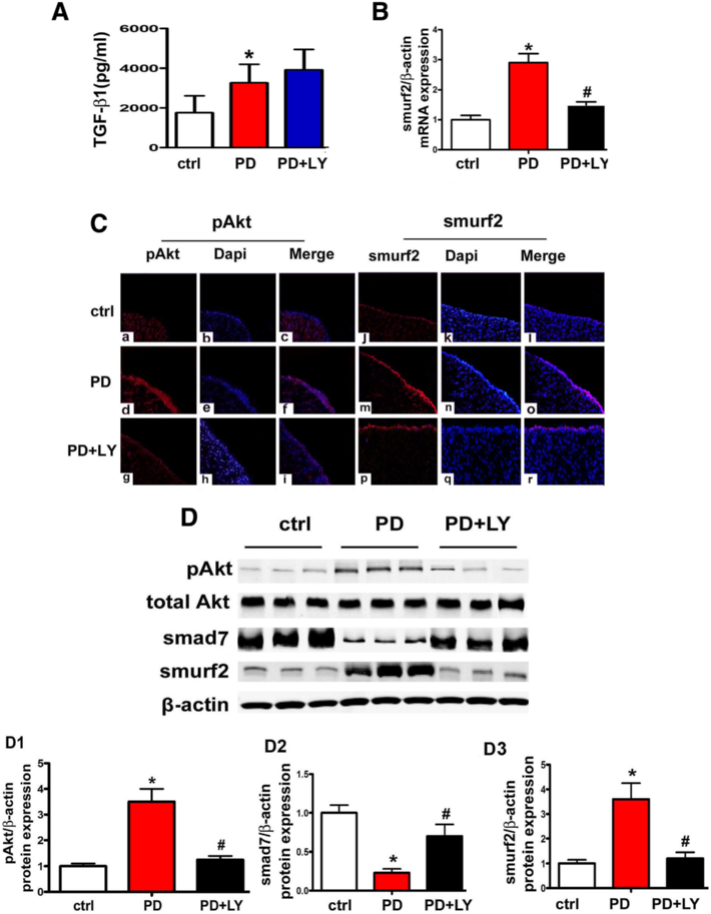
**Expression of TGF-β1, pAkt, Smurf2 and Smad7 in mice undergone peritoneal dialysis (PD) and the modulation of Smurf2 and Smad7 expression by PI3/Akt inhibitor (LY294002). Panel A**: Bar graphs represent the summary of TGF-β1 concentration in the PD effluent in each group, as measured by ELISA method. **Panel B**: Real-time PCR showing increased mRNA expression of Smurf2 in the peritoneal tissue of PD mice, whereas it was inhibited by the treatment with PI3/Akt inhibitor LY294002. **Panel C**: Immunofluorescence microscopy of peritoneal tissue stained with either specific anti-pAkt or anti-Smurf2 antibodies (red) and counter stain with DAPI (blue). Increased immuno-reactive signals representing expression of pAkt and Smurf2 were observed in the peritoneum of PD mice and were markedly decreased with the LY294002 treatment (original magnifications × 200). **Panel D**: The expression of pAkt, Smad and Smurf2 in the peritoneal tissue of PD mice, as assessed by Western blot analyses. Panels D1-D3: The bar graphs represent the densitometric measurements of Western blot bands. Values are the mean ± SEM, n = 5, * p <0.01 vs. control, # p <0.01 vs. PD group.

ELISA assay showed that the concentration of TGF-β1 increased in the peritoneal effluent of PD mice compared to control (P < 0.01), while there are no further significant changes in mice treated with the PI3K/Akt inhibitor, LY294002 (Figure 1A). By real-time PCR, an up-regulated expression of smurf2 mRNA was observed in the peritoneal tissues of PD mice, while it was dramatically down-regulated in mice treated with LY294002 (Figure 1B). Confocal imaging with Anti-phospho-Akt1 (Ser473/Tyr474) antibody (anti-pAkt) showed that there was a low level of phosphorylated Akt (pAkt) expression in the peritoneum of control mice and it markedly increased in PD mice. The expression was significantly inhibited by LY294002 (Figure 1C, left panels). Like the pAkt, a parallel increase in the Smurf2 levels was also observed (Figure 1C, right panels). In addition, Western blot analyses revealed that the protein expression of pAkt (Figure 1D, upper panels, D1) and Smurf2 (Figure 1D, lower panels, D3) were markedly increased in PD mice compared to control, which was reversed by treatment with LY294002. In contrast, a decreased expression of Smad7 protein was seen in PD mice, and Smad7 expression was restored by the treatment with LY294002 (Figure 1D, middle panels, D2).

### Inhibition of Akt normalizes the altered expression of ZO-1, Vimentin and fibrosis in the peritoneum of PD mice

**Figure 2:**
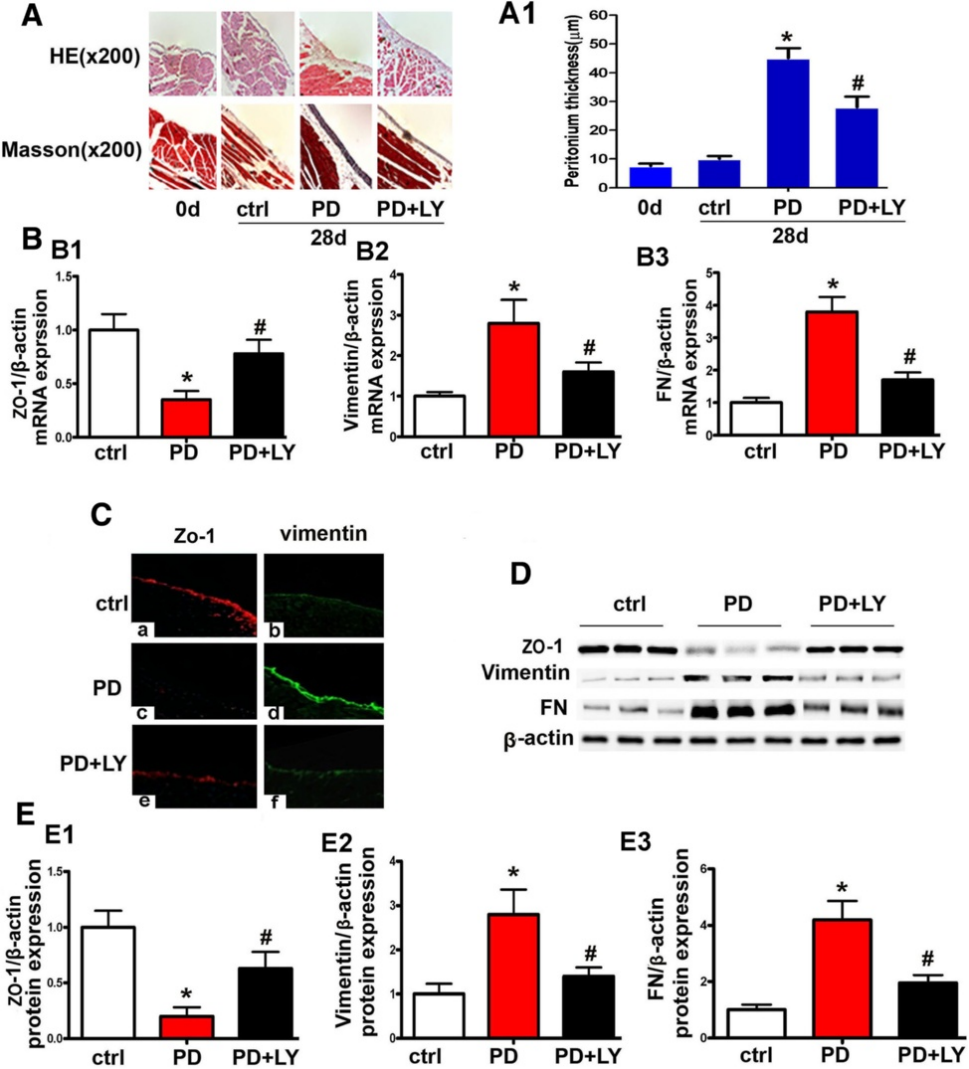
**PI3K/Akt inhibitor normalized MMT relevant proteins’ expression and peritoneal fibrosis in PD mice model. Panel A**: Hematoxylin-Eosin (top panels) and Masson’s Trichrome staining (bottom panels) of the parietal peritoneum in control mice, PD mice with or without LY294002 treatment. Panel A1: Semi quantification of H & E staining for the thickness of the sub-mesothelial tissue of the parietal peritoneum. **Panels B1 - B3**: Bar graphs represent the mRNA expression of ZO-1, Vimentin and fibronectin (FN) in peritoneal tissues, as detected by real-time PCR. **Panel C**: Immunofluorescence microscopy of the peritoneal tissue sections following staining with ZO-1 (red) and Vimentin (green) antibodies. Treatment with LY294002 restored the expression of ZO-1 to a certain extent and inhibited the over-expression of Vimentin in the peritoneum compared to PD mice. **Panel D**: Western blot analysis showed the expression of ZO-1, Vimentin and FN. Panels D1-D3. **Panels E1-E3**: Bar graphs represent the densitometric measurements of the bands seen by Western blot analyses. Values are the mean ± SEM, n = 6, * p <0.01 vs. control group, # p <0.01 vs. PD group.

Morphological changes in the peritoneum were assessed by H & E and Masson staining. The thickness of the sub-mesothelial tissue in the peritoneum of PD mice increased approximately 4–5 times compared to control, whereas, it was dramatically attenuated in mice treated with LY294002 (Figure 2A and 2A1). In addition, by real time PCR, Vimentin and fibronectin (FN) mRNA expression in the peritoneal tissue of PD mice were significantly increased, which was accompanied with markedly decreased mRNA expression of ZO-1. However, treatment with LY294002 led to a significant normalization of their expression (Figure 2B, B1-B3). Similar results were observed for the protein expression by tissue image analysis with immunofluorescence microscopy (Figure 2C), which were confirmed by Western blot analyses (Figure 2D and 2E, E1-E3).

### TGF-β1 increases the expression of pAkt, Smurf2 and MMT-relevant proteins and down-regulates Smad7 expression in HPMCs

**Figure 3:**
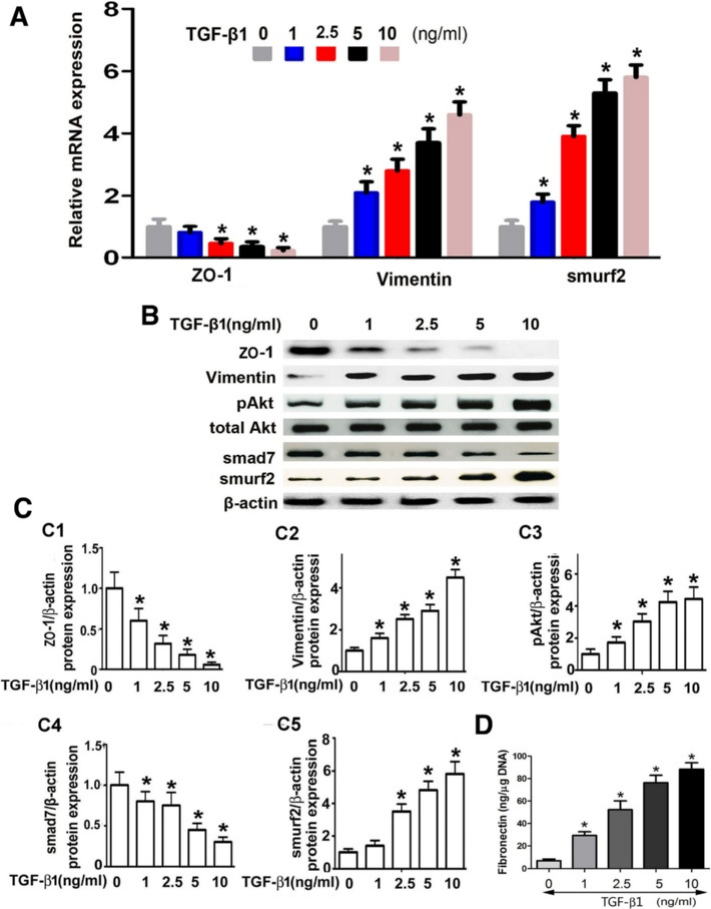
**Expression of EMT/MMT relevant protein, fibronectin, smurf2, Smad7 and pAkt in HPMCs following TGF-β** **1 treatment. Panel A**: By Real-time PCR, a dose-dependent decrease in mRNA expression of ZO-1 was seen in HPMCs treated with TGF-β1, while an increased expression of Vimentin and Smurf2 miRNA was observed. **Panel B**: Western blot analyses showed that the protein expression of ZO-1 and Smad7 was decreased with a dose-dependent manner in HPMCs treated with TGF-β1. In contrast, the expression of Vimentin, pAkt and Smurf2 was significantly increased. **Panel C**: The bar graphs represent the densitometric measurements of the bands seen by Western blotting procedures. **Panel D**: The bar graph represents the fibronectin concentration in the supernatant of cultured HPMCs induced by TGF-β1 and detected By ELISA. Values are the mean ± SEM, n = 6, * p <0.01 vs. control.

HPMCs were exposed to different concentrations of TGF-β1 (0–10 ng/ml) for 48 hrs. By real time PCR, a significant dose dependent decrease in the mRNA expression of ZO-1 was observed. In contrast, the mRNA expression of Vimentin and Smurf2 were increased markedly in a dose dependent manner (Figure 3A). Western blot analyses showed that a dose-dependent up-regulated expression of pAkt and Smurf2 in HPMCs following stimulation with TGF-β1, while the expression of Smad7 protein was significantly down-regulated. In addition, a decreased expression of ZO-1 and an increased expression of Vimentin in a dose-dependent manner were observed (Figure 3B and 3C, C1-C5). Furthermore, by ELISA, a dose-dependent increased protein concentration of FN was noted in HPMCs exposed to TGF-β1 (Figure 3D).

**Figure 4:**
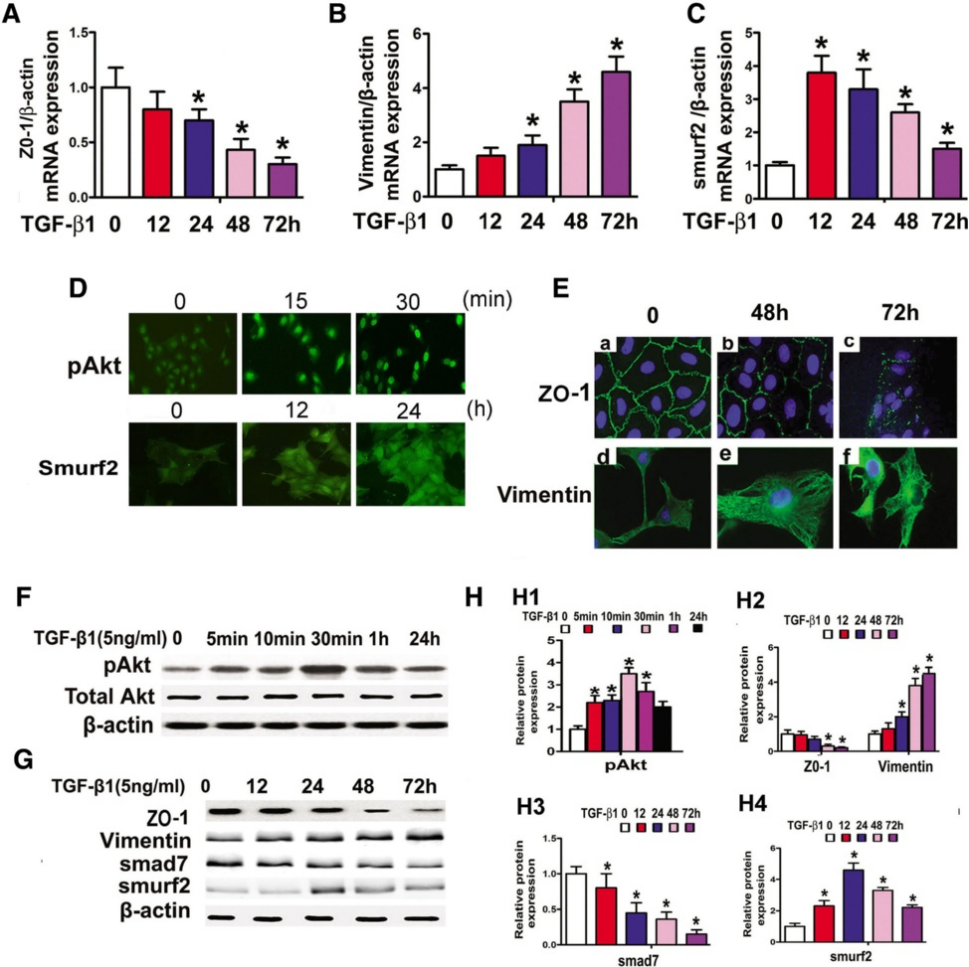
**Effect of TGF-β1 on the expression of pAkt, ZO-1, Vimentin, Smurf2 and Smad7 in HPMCs, as assessed by Real-time PCR (Panels A-C), Cell Immuno- fluorescence (Panels D and E) and Western blot assay (Panels F, G & H1-H4).** The bar graphs show a time-dependent mRNA expression of ZO-1 (**Panel A**), Vimentin (**Panel B**) and Smurf2 (**Panel C**) in HPMCs after treatment with TGF-β1 (5 ng/ml) for 0–72 hrs. **Panels D** and **E**: Confocal images of cells after staining with anti-pAkt, −Smurf2, −ZO-1 and -Vimentin antibodies. An increased nuclear expression of pAkt was observed in HPMCs following treatment with 5 ng/ml of TGF-β1 for 30 min (Panel D, upper panels). The Smurf2 expression in cytoplasm was also significantly increased (Panel D, lower panels). The ZO-1 expression was decreased in a time-dependent manner in HPMCs following treatment with TGF-β1 for 48 hr, which further decreased at 72 hr (Panel E, upper panels), In contrast, Vimentin expression was increased (Panel E, lower panels). **Panel F** & **G**: Western blot analyses of protein extract from HPMCs showed that pAkt and smurf2 expression initially increased in HPMCs, and then gradually decreased. While ZO-1 and Smad7 expression was gradually decreased over time. The Vimentin expression was significantly increased gradually over time. Panel H: The bar graphs represent the band density of Western blots. Values are the mean ± SEM, n = 3, * p <0.01 vs. control.

To delineate the role of TGF-β1 in the gene and protein expression of above indicated molecules with time course, HPMCs were incubated with 5 ng/ml TGF-β1 for 0–72 hrs. As shown in Figure 4A-4C, ZO-1 mRNA expression was significantly decreased in a time dependent manner (Figure 4A), while that of Vimentin expression was increased (Figure 4B). Interestingly, the smurf2 expression showed a dramatically increase up to 12–48 hrs and then it was somewhat reduced but did not reach to the basal levels (Figure 4C). Confocal image of cell immunofluorescence clearly showed an enhanced pAkt nuclear expression in HPMCs after incubation with TGF-β1 for 15 min, and it increased a little bit further at 30 min (Figure 4D, upper panels). The expression of Smurf2 was also increased in the cytoplasm of HPMCs treated by TGF-β1 for 12 hrs, and it peaked at 24 hrs (Figure 4D, lower panels). Additionally, a significant decreased immunofluorescence signal in the cell membrane, depicting ZO-1 expression, was observed in HPMCs treated with TGF-β1at 48 hrs, and it further decreased at 72 hrs (Figure 4E, upper panels). The Vimentin expression increased in a time-dependent manner (Figure 4E, lower panels). By Western blot analyses, pAkt protein expression was increased significantly in HPMCs treated with TGF-β1 for 30 min, and then it gradually decreased but did not reach to the basal levels (Figure 4F, upper panels and 4H, H1). Similar expression profiles were also observed for the Smurf2 protein where it peaked at 24 hrs (Figure 4G, lower panels and 4H, H4). The ZO-1 and Smad7 expression was significantly decreased in a time-dependent manner up to 72 hrs (Figure 4G and 4H, H3); and in contrast, Vimentin expression was increased (Figure 4G, middle panels and 4H, H2).

### Akt inhibited TGF-β1-induced expression of MMT relevant proteins in HPMCs

**Figure 5:**
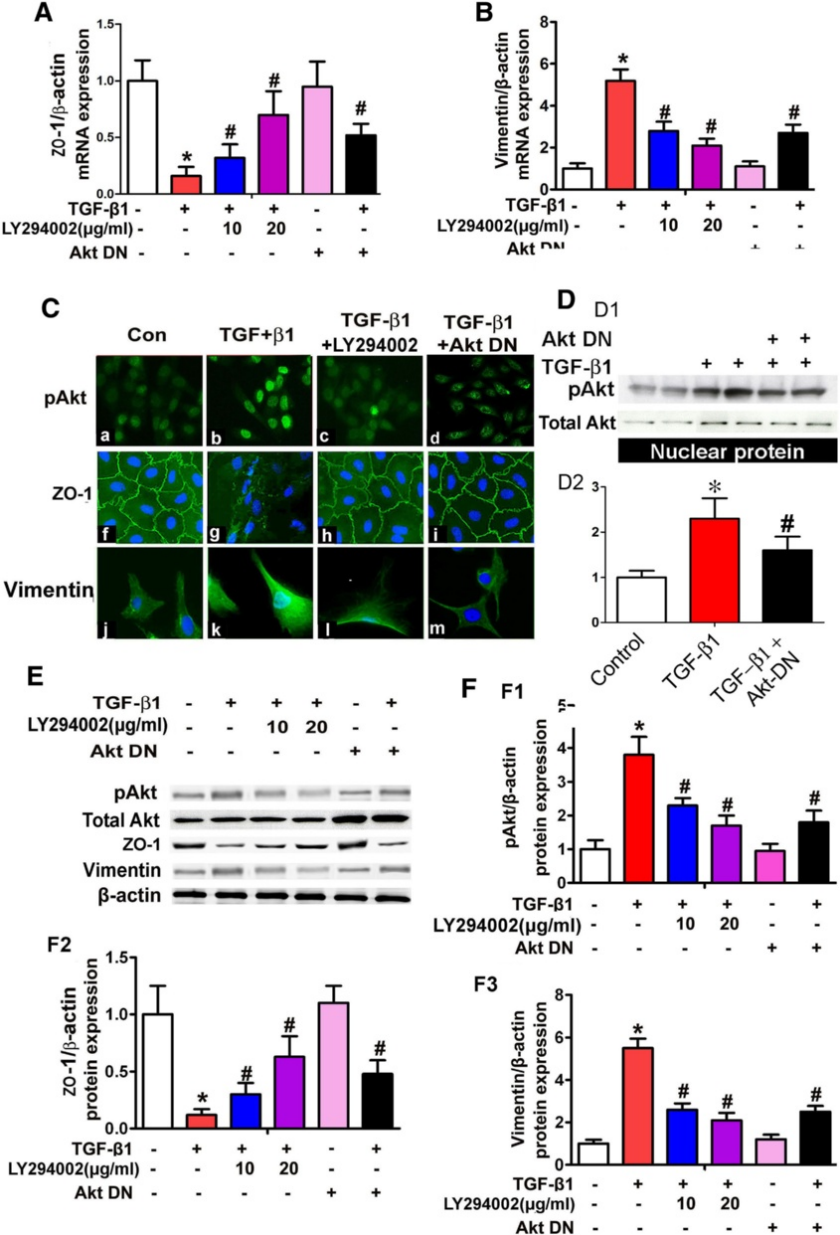
**Effect of Akt-DN and LY294002 on ZO-1 and Vimentin expression in HPMCs. Panels A** and **B** represent summary of Real-time RT-PCR. A reduced mRNA expression of ZO-1 (Panel A) and increased that of Vimentin (Panel B) in HPMCs stimulated with TGF-β1 was observed, while the expression was partially restored to pre-treatment levels with the PI3K inhibitor, LY294002 or by transfection of dominant-negative Akt (Akt-DN) plasmid. **Panel C**: By immunofluorescence microscopy, down-regulated ZO-1 and up-regulated Vimentin expression was observed in HPMCs subjected to TGF-β1 treatment, and it was normalized with transfection with Akt-DN or pretreatment with LY294002. On the other hand, TGF-β1 induced pAkt translocation to the nucleus in HPMCS, while partially blocked by treatment with either Akt-DN and LY294002. Similar results were also observed in nuclear extract from HPMCs, as detected by Western blot analyses (**Panel D**, D1-D2). **Panel E**: Western blot analyses showed down- regulated ZO-1 expression in HPMCs stimulated with TGF-β1, while partially reversed in that of treatment with LY294002 or Akt-DN plasmid. In contrast, an up-regulated Vimentin and pAkt expression was seen in HPMCs stimulated with TGF-β1 and partial normalization following the treatment with LY294002 or Akt-DN plasmid. **Panels F**, F1-F3: Bar graphs represent the density of relative bands of Western blots. Values are the mean ± SEM, n = 3, * p <0.01 vs. control, # p <0.01 vs.TGF-β1.

Real- time PCR showed that ZO-1 mRNA expression decreased significantly in HPMCs treated with TGF-β1, while it was partially restored by the treatment with LY294002 or by transfection of with an Akt-dominant negative plasmid (Akt-DN) (Figure 5A), In addition, opposite results were observed for Vimentin mRNA expression (Figure 5B). By immunofluorescence microscopy, HPMCs stained with anti-pAkt, −ZO-1, −Vimentin antibodies yielded parallel results. TGF-β1 significantly increased the expression of pAkt, while it was reversed with the treatment of LY294002 or over-expression of Akt-DN (Figure 5C upper panels). Additionally, cells treated with LY294002 or Akt-DN had normalized altered ZO-1 and Vimentin expression in HPMCs concomitantly treated with TGF-β1 (Figure 5C middle and lower panels). Furthermore, by Western blot analysis, an increased expression of pAkt in the nuclear extract from HPMCs treated with TGF-β1 was observed, while it was reduced in cells transfected with Akt-DN plasmid, while no significant change was seen in the total Akt expression (Figure 5D). On the other hand, pAkt expression was decreased in cytoplasmic extract from HPMCs treated with TGF-β1, while normalized following treatment with LY294002 or Akt-DN (Figure 5E, upper panels and 5F, F1), as detected by Western blot analyses. Similar results were also observed in the EMT/MMT relevant proteins such as Vimentin expression (Figure 5E, lower panels and 5F, F3). In contrast, ZO-1 protein expression was noted to be decreased in HPMCs treated with TGF-β1. However, the effect was partially blocked by the treatment with LY294002 or Akt-DN (Figure 5E, middle panels, and 5F, F2).

### Effect of Akt on the expression of Smurf2 and Smad7

**Figure 6:**
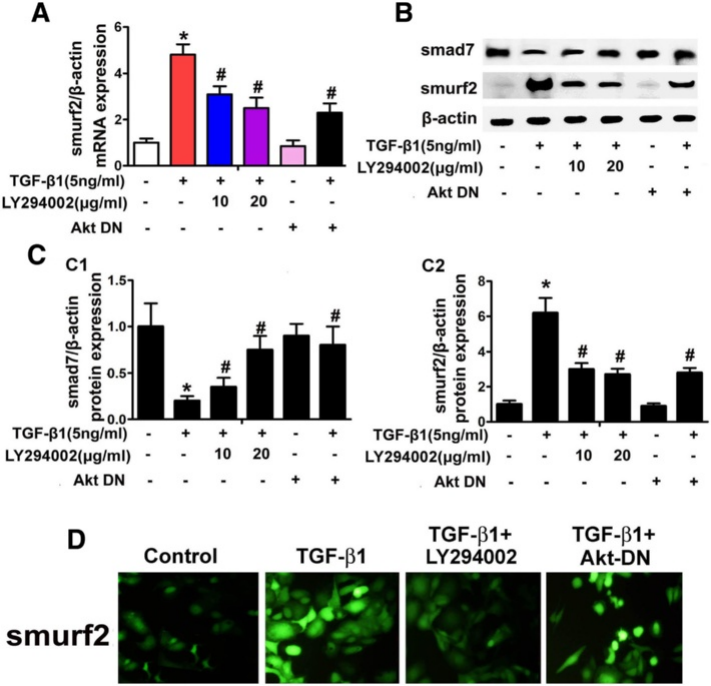
**Expression of Smurf2 and Smad7 in HPMCs following exposure to TGF-β** **with/without treatment of LY294002 and Akt-DN. Panel A**: Bar graph represents the summary of Smurf2 mRNA expression, as detected by Real-time PCR analyses. **Panel B**: Represents of Smurf2 and Smad7 expression profiles following treatment of LY294002 or transfected with Akt-DN, as assessed by Western blot analyses. The β-actin served as a loading control. **Panel C**: The bar graphs represent the relative bands density of Smad7 (Panel C1) and Smurf2 (Panel C2) of Western blots. A decreased expression of Smad7 was observed in HPMCs incubated with TGF-β1, while the effect was abolished by the pre-treatment with LY294002 and transfection with Akt-DN, in addition, TGF-β1 significantly up-regulated the expression of Smurf2, which was inhibited with Akt-DN or LY294002. **Panel D**: Immunofluorescence microscopy revealed that TGF-β1 significantly increased Smurf2 expression in HPMCs, which was dramatically decreased by LY294002 (next to the extreme right panel), but only partially attenuated by transfection with Akt-DN (extreme right panel). Values are the mean ± SEM, n = 5, * p <0.01 vs control, # p <0.01 vs TGF-β1.

Real-time PCR revealed that mRNA expression of Smurf2 significantly increased in HPMCs treated with TGF-β1, while the expression was blocked partially in a dose-dependent manner either by the treatment with LY294002 or transfection with Akt-DN mutant plasmid (Figure 6A). Western blot analyses showed that the Smad7 protein expression was significantly inhibited in HPMCs incubated with TGF-β1, while it was restored partially by the treatment with either LY294002 or transfection with an Akt-DN (Figure 6B and 6C, C1). In contract, the Smurf2 expression was increased in HPMCs treated with TGF-β1, while it was reduced in cells treated with Y294002 or transfection with an Akt-DN (Figure 6B and 6C, C2). In addition, the up-regulated expression of Smurf2 was confirmed by immunofluorescence microscopy following the staining with anti-Smurf2 antibody. Interestingly, Akt-DN only partially inhibited the TGF-β-induced Smurf2 expression (panel on the extreme right, Figure 6D) while completely with LY294002 (panel next to TGF-β1, Figure 6D).

### Effect of Akt, Smurf2 and USP4 on the expression of ZO-1, Vimentin and Fibronectin in HPMCs incubated with TGF-β1

**Figure 7:**
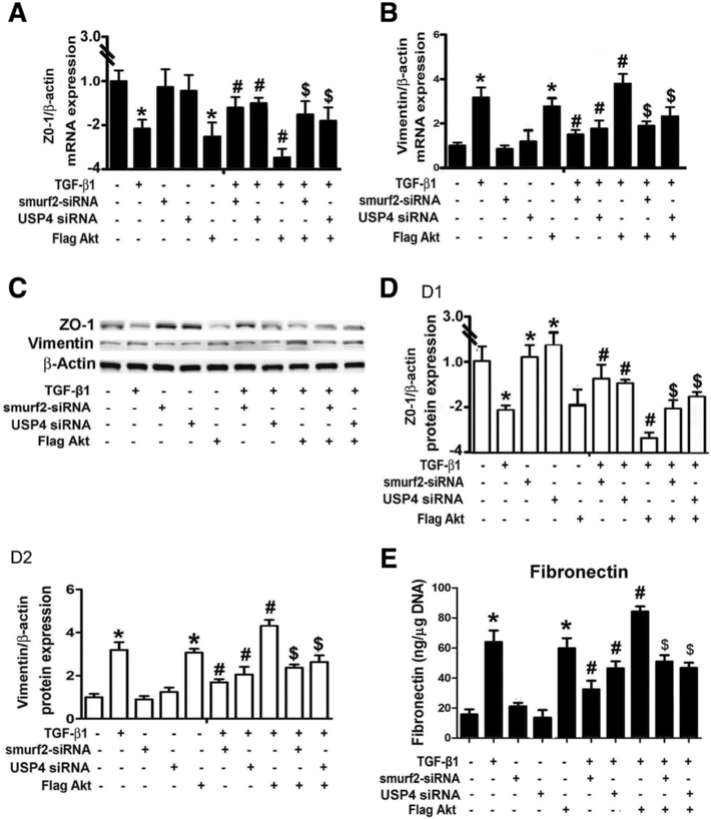
**Role of Smurf2 and USP4 in TGF-β** **1 or Akt-mediated ZO-1, Vimentin and Fibronectin expression in HPMCs. Panels A** & **B**: The bar graphs represent the summary of the mRNA expression of ZO-1 and Vimentin, as detected by Real-Time PCR. **Panel C**: Western blot analyses indicated a decreased protein expression of ZO-1 in HPMCs treated with TGF-β1 or co-transfected with Flag-Akt. There was no obvious change in cells treated with Smurf2-siRNA and USP4 siRNA compared to basal levels. The TGF-β1-mediated reduced expression of ZO-1 was partially reversed with the transfection of Smurf2-siRNA and USP4 siRNA. ZO-1 expression was markedly decreased by the treatment with TGF-β1 and transfection with Flag-Akt. The Smurf2-siRNA and USP4 siRNA also partially restored the TGF-β1 + Flag-AKT reduced ZO-1 mRNA expression. **Panel D**, D1-D2: The bar graphs represent the Western blot relative band density of ZO-1 and Vimentin compared to β-actin. **Panel E**: Bar graphs represent the fibronectin concentration in the supernatant of cultured HPMCs, as detected by ELISA. Values are the mean ± SEM, n = 4, *p < 0.01 vs control. # p <0.01 vs TGF-β1, $ p <0.01 vs TGF-β1+ Flag-Akt.

By real-time PCR, a significantly decreased mRNA expression of ZO-1 was seen in HPMCs incubated with TGF-β1 or transfected with Flag-Akt plasmid (Figure 7A, lanes 2 and 5 vs lane 1), while no affect was seen in cells treated with Smurf2 siRNA and USP4 siRNA (Figure 7A, lanes 3, 4 vs lane 1). TGF-β1 reduced ZO-1 mRNA expression, which was restored following concomitant treatment with Smurf2 siRNA and USP4 siRNA (Figure 7A, lanes 6 and 7 vs lane 2). In addition, ZO-1 expression was decreased by TGF-β1 plus Flag-Akt treatment (Figure 7A, lane 8 vs lane 2), and it was partially restored by transfection of Smurf2 siRNA or USP4 siRNA (Figure 7A, lanes 9 and 10 vs lane 8). In contrast, opposite results were observed for the Vimentin mRNA expression (Figure 7B). Similar results were also seen for the protein expression of ZO-1 and Vimentin, as detected by Western blot procedures (Figure 7C and 7D, D1 and D2). Fibronectin (FN) protein expression in HPMCs was also measured by ELISA. An increased FN expression was seen in HPMCs exposed to TGF-β1 or transfected with Flag-Akt (Figure 7D, lanes 2 and 5 vs lane 1). No change in the FN expression was observed in cells treated with Smurf2 siRNA or USP4 siRNA alone compared to basal levels. The effect of TGF-β1 on the expression of FN was partially blocked by the treatment with Smurf2 siRNA or USP4 siRNA (Figure 7D, lanes 6 and 7 vs lane 2), but was further augmented with transfection of Flag-Akt. In addition, Smurf2 siRNA and USP4 siRNA reduced the up-regulated expression of FN in HPMCs induced by TGF-β1plus Flag-Akt (Figure 7D, lanes 9 and 10 vs lane 8). Compared to controls, the mRNA and protein expression did not change in cells treated with scrambled siRNAs (data not shown - space limitations).

### Effect of TGF-β1, Akt and USP4 on TβR-1, Smad7 and smurf2 expression and the interaction between Smurf2 and Smad7 as well as TβR-1

**Figure 8:**
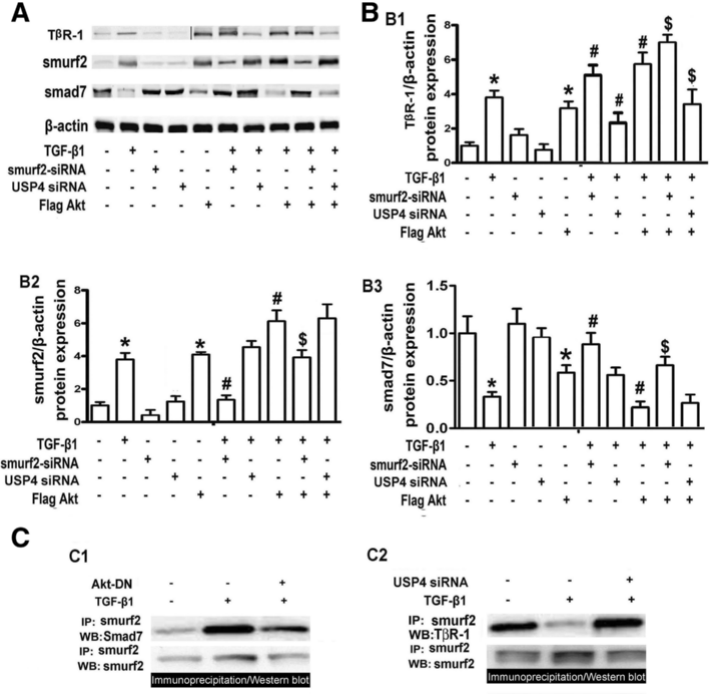
**Expression of T** **β** **R-1, Smad7 and Smurf2 in HPMCs stimulated by TGF-** **β** **1 and treated with Akt, Smurf siRNA and USP4 siRNA. Panel A**: Western blot profiles of TβR-1, Smad7 and Smurf2 following various treatments as indicated. The β-actin served as a loading control. **Panel B**: The bar graphs represent the relative density of the TβR-1 (B1), Smurf2 (B2) and Smad7 (B3) Western blots bands relative to β-actin. Treatment of TGF- β1 or over-expression of Akt (Flag-Akt) enhances the TβR-1 and Smurf2 protein expression, while it reduces the Smad7 expression. A concomitant treatment and over-expression further enhances their expression. No change is seen in cells treated with Smurf2 siRNA or USP4 siRNA alone. The TGF-β1+ Flag Akt-induced Smurf2 expression was slightly reduced by Smurf2-siRNA and not by USP4-siRNA. The Smad7 expression is reduced with TGF-β1 or Flag-Akt individually, and it is slightly reversed by Smurf2 siRNA. **Panel C**: Immunoprecipitation/Western blot analyses represent changes seen in interactions between Smurf2 & Smad7, and Smurf2 & TβR-1 (upper panels). The Smurf2 expression served as loading controls (bottom panels). Values are the mean ± SEM, n = 3, *p <0.01 versus control, #p <0.01 vs. TGF-β1, $p <0.01 vs. TGF-β1+ Flag-Akt.

Western blot analyses showed that the TβR-1 expression was significantly increased in HPMCs treated with TGF-β1 and Flag-Akt (Figure 8A and 8B, B1, lanes 2 & 5 vs lane 1), whereas no change was seen in cells treated with Smurf2 siRNA or USP4 siRNA (Figure 8A and 8B, B1, lanes 3 & 4 vs lane 1). TGF-β1 up-regulated expression of TβR-1 was further enhanced by treatment with Smurf2 siRNA and Flag-Akt plasmid (Figure 8A and 8B, B1, lanes 6 & 8 vs lane 2), while it was reduced by USP4 siRNA (lane 7 vs lane 2). In addition, TGF-β1 plus Flag-Akt increased TβR-1 expression was augmented with the co-transfection of Smurf2 siRNA (Figure 8A top panels and 8B, B1, lane 9 vs lane 8). It was also noted that Smad7 protein levels decreased in HPMCs treated with TGF-β1 or Flag-Akt plasmid. No change was seen in Smurf2 siRNA or USP4 siRNA. TGF-β1 decreased Smad7 expression, and it was partially reversed by the pre-treatment of smurf2 siRNA or USP4 siRNA. The TGF-β1 decreased Smad7 expression was little bit further reduced by co-transfection with Flag-Akt. TGF-β1 plus Flag-Akt reduced Smad7 expression, and it was partially blocked by either co-transfection with Smurf2 siRNA or USP4 siRNA (Figure 8A, and 8B, B2). In addition, there were no significant differences in Smurf2 expression between cells treated with TGF-β1+ Flag-Akt and TGF-β1+ Flag-Akt + USP4 siRNA. Almost opposite results were seen for Smad7 protein expression compared to the Smurf2 expression profiles following various treatments (Figure 8A middle panels, and 8B, B3).

The immunoprecipitation/Western blot (IP/WB) assay revealed an increased signal representing the interaction between Smurf2 and Smad7 in HPMCs following TGF-β1 treatment (Figure 8C, C1, lane 2 vs lane 1). However, the signal was significantly reduced with the transfection of Akt-DN (lane 3 vs lane 2). On the other hand, TGF-β1 reduced the association between Smurf2 and TβR-1, while it was restored by treatment with USP4 siRNA (Figure 8C, C2, upper panels). Smurf2 expression by IP/WB revealed no significant change, and Smurf2 served as loading control (Figure 8C, C1 & C2, lower panels).

## Discussion

The observations made in this investigation suggest a relevance of Akt with respect to the pathology of Mesothelial Mesenchymal Transition/Transformation (MMT) in human peritoneal mesothelial cells (HPMCs) and peritoneal fibrosis in mice undergoing peritoneal dialysis (PD). We demonstrated that Akt modulates TGF-β1-induced MMT and peritoneal fibrosis in PD *via* downstream interactive signaling molecules, such as, Smurf2 and Smad7, and USP4 and TβR-1.

Epithelial-Mesenchymal Transition/Transformation (EMT), a prototype of MMT, is a complex and generally reversible process that plays an important role in embryogenesis, metastasis of malignant cells and tissue fibrosis [23,24]. During peritoneal dialysis, mesothelial cells undergo EMT/ MMT associated with peritoneal-membrane injury. Conceivably, blocking MMT by Tamoxifen in the PD animal model can ameliorate peritoneal membrane damage [25]. MMT could lead to peritoneal fibrosis and peritoneal membrane dysfunction during long-term PD [26]. Recent studies have shown that a large number of factors involved in the process of MMT in PD. Consensus from various studies are that TGF-β1 is a critical inducer in MMT and fibrosis in PD [27]. Previously, we demonstrated that TGF-β1 induced EMT in human peritoneal mesothelial cells (HPMCs) [28]. Here, we extended our studies in animal models and demonstrated that MMT is associated with some of the ECM proteins, such as fibronectin and fibrosis in the peritoneum of mice undergoing PD (Figure 2A-D). Consistent with this was thickening of sub-mesothelial tissues and up-regulation of TGF-β1 in PD effluent (Figure 1A). In addition, the *in vitro* studies confirmed that TGF-β1 is capable of inducing the MMT-relevant proteins expression in HPMCs (Figures 3A, 3B, 4A, 4B, 4D and 4E). The expression of these proteins seems to be modulated by increased or reduced expression of Akt with TGF-β1 being the upstream regulator. Our results are consistent with previous reports indicating inhibiting TGF-β1 signaling ameliorates experimental peritoneal fibrosis [29]. These studies also implicated that Akt could play a critical role in TGF-β1 induced MMT and peritoneal fibrosis in PD, although detailed mechanisms pertaining to mesothelial pathobiology remained to be work out. Much of the work in this regard has been focused on tubular epithelial cell biology.

Emerging evidence shows that the PI3K/Akt pathway is another non-Smad pathway contributing to TGF-β induced EMT [30]. PI3K/Akt modulates TGF-β1-induced EMT in renal tubular cells [12]. TGF-β apparently causes phosphorylation of Akt, which seems to be required for TGF-β-induced EMT [31]. Moreover, LY294002, an inhibitor of PI3K/Akt, can attenuate insulin-induced PI3 kinase-dependent Akt phosphorylation and reduce apoptosis in peritoneal mesothelial cells [32]. Treatment with LY294002 also significantly reduces TGF-β1-stimulated COL1A2 promoter activity and expression, as well as diminishes the effect of VEGF and thus conceivably inhibits EMT transformation in renal tubular epithelial cells [33,34]. Furthermore, TGF-β induced down-regulation of E-cadherin in renal tubular epithelial cells has been reported following the treatment with LY294002, suggesting that Akt may play a key role in TGF-β induced EMT transformation [35]. In addition, PI3K/Akt pathway regulates TGF-β signaling cascade by inducing Smad7 expression [36]. However, whether Akt is involved in the process of TGF-β induce MMT in PD by modulating the pathobiology of mesothelial cells is uncertain. Also, the mechanisms by which Akt mediates MMT coupled fibrosis in PD or by TGF-β1 stimulating peritoneal mesothelial cells have not been so far clearly delineated.

Certainly, it is known that TGF-β family members transduce signals through membrane serine/threonine kinase receptors and intracellular Smad proteins including Smad2/3 which are regulated by Smad7 *via* E3 ubiquitin ligases, such as, Smurf2 [37]. In addition, TGF-β stimulates Smurf2 promoter activity and its expression by a Smad-independent pathway, such as, the PI3K/Akt pathway [13]. Smurf2 contains three WW domains, which can bind to the Smad7 PY motif region and form a complex [38]. After Smurf2 binding to Smad7 it is exported to the membrane of cells, where it causes degradation of Smad7 and TGB-β1 receptor [39]. Furthermore, in the presence of TGF-β Signaling the degradation of Smad7 is dependent on the catalytic activity of the Smurf2 HECT domain [40]. Recently, Tan RY *et al*. found that Smurf2 expression is significantly increased upon TGF-β1 stimulation, and over-expression of Smurf2 augmented TGF-β1-mediated E-cadherin suppression in human tubular epithelial cells [41]. On the other hand, Smad7 negatively regulates the TGF-β1/Smad pathway [42,43]. Overexpression of Smad7 has been shown to inhibit AngII-induced TGF-β/Smad activation and EMT in NRK52E cells and in a rat model of remnant kidney disease [44]. Interestingly, transfer of Smad7 gene by ultrasound microbubble method into the peritoneum inhibits Smad2/3 activation, decreases α-SMA expression, and attenuates peritoneal fibrosis in a rat model of PD [45]. Taken together the data of above indicated studies, it seems that the activation of TGF-β1-Akt-Smurf2-Smad7 pathway may mediate MMT in PD. Thus, we hypothesized that activation of Akt by TGF-β1 increases Smurf2 expression which would inhibit Smad7, and thus Akt could participate in TGF-β1-induced MMT and peritoneal fibrosis in mice undergoing PD.

To address all these myriad of issues, *in vivo* and *in vitro* studies were carried out in PD mice model and HPMCs by the multitude of methods, including tissue morphology, Real Time PCR, immunohistochemistry and Western blot analyses. We observed that the expression and activity of pAkt and Smurf2 were increased in the peritoneum of PD mice while Smad7 was decreased (Figure 1B-1C). In addition, pAkt expression rapidly increased in the nuclear compartment in a dose-dependent manner in HPMCs subjected to TGF-β1 (Figure 4D and 4F), which was accompanied with altered of Smurf2, Smad7 and MMT-relevant proteins’ expression (Figure 4G and 4H). The altered expression of Smurf2, Smad7 and EMT relevant proteins in the peritoneum of PD mice was reversed following *in vivo* treatment with LY294002 (Figure 1D), and so was case when TGF-β1-induced HPMCs were treated *in vitro* with LY294002 or transfected with Flag-Akt or Akt-DN expression plasmids (Figures 5, 6 and 7). These observations clearly indicate that the activation of pAkt induces MMT transformation during PD and it mediated *via* the increased expression of Smurf2 with simultaneous down-regulation of Smad7.

The next question that needs to be addressed pertains to the status of TGF-β1 receptor during MMT transformation. TGF-β signaling pathway is tightly regulated through protein ubiquitination, *e.g.*, TRβ1 degradation. It is known that levels of TβRI at the cell surface are regulated by Smurf 2/Smad7 complex, and the stability of this receptor determines the status of TGF-β signaling [40]. In this regard, it has been shown that the treatment with TβR-I inhibitor suppresses the lymphangiogenesis and VEGF-C expression in a murine model of peritoneal fibrosis [46]. With respect to TβR-I pathobiology, extensive studies carried out in the past in a vast number of systems indicate that there is a crosstalk between PI3K/Akt and TGF-β signaling pathways, which at times counteract while in other situations cooperate with one another [47]. Recently Eichhorn *et al*. using a functional RNAi screen identified the deubiquitinating enzyme ubiquitin-specific protease 15 (USP15) as a key component of the TGF-β signaling pathway [21]. USP15 binds to Smurf2 complex and deubiquitinates and stabilizes TRβ1, and that apparently leads to boosting of TGF-β-mediated signaling. Likewise USP4 as a deubiquitylating enzyme also interacts with TβRI at the plasma membrane and reverses its ubiquitination and thus regulates the TβRI levels [16]. Conceivably, AKT directly interacts with USP4 for its phosphorylation and translocates nuclear USP4 to the membrane and thus maintains TβRI stability and possibly modulates TGF-β induced EMT/MMT.

In reference to the role of Smurf2 and USP4 in MMT in PD. our data indeed show that there is some modulation by the Akt in downstream signaling. To verify the effect of Akt regulation on MMT in PD, Smurf2 siRNA and USP4 siRNA experiments were carried out in HPMCs. Results showed that inhibition of USP4 and Smurf2 expression significantly reverses the altered expression of ZO-1, Vimentin and TβRI induced by TGF-β1, while overexpression of Akt augments these effects. Transfection with USP4 siRNA or Smurf2 siRNA also inhibited the effect exerted by TGFβ1+ Akt on the expression of TβRI, ZO-1 and Vimentin (Figures 7 and 8). Additionally, to address the issue that whether Akt and USP4 regulate the interaction between Smurf2 and Smad7, and Smurf2 and TβRI induced by TGF-β1, immunoprecipitation/Western blot assays were carried out. They revealed that Akt-DN and USP4 siRNA can disrupt their association in HPMCs treated with TGF-β1 (Figure 8D). This indicated that Akt may regulate MMT in PD *via* various signaling pathways which involve the Smurf2/USP4/TβRI complex.

## Conclusions

**Figure 9:**
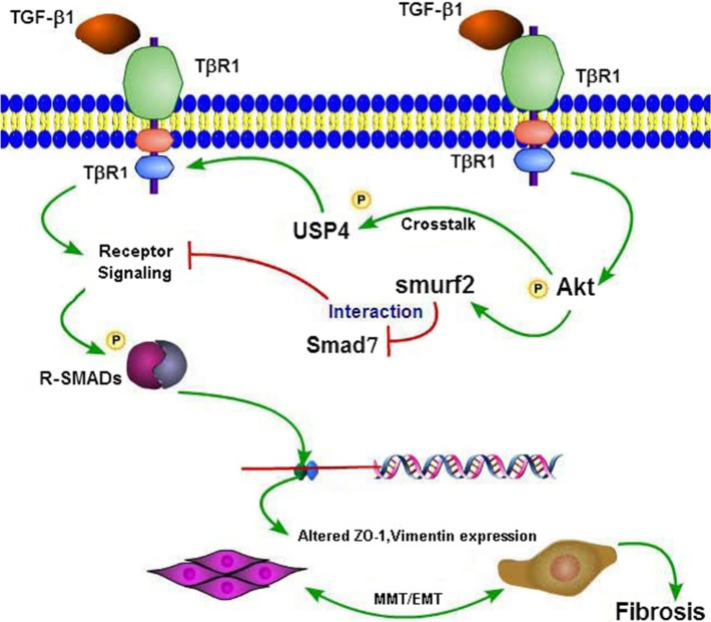
**Schematic drawing depicting the conceivable events following TGF-β1 treatment and activation of Akt.** This leads to initiation of transcription of Smuf2 and degradation of Smad7 and phosphorylation of USP4 while maintaining the TβRI stability. Finally, this results in the induction of MMT of mesothelial cells and fibrosis during peritoneal dialysis.

The findings of the current study highlights the activation of Akt mediated EMT/MMT transformation in PD *via* Smurf2 modulation/and or Smad7 degradation while conceivably maintaining the TβRI stability, most likely by the USP4. The findings also suggest that Akt plays a central role in MMT and fibrosis during PD through Smurf2/Smad7 and USP4/TβRI pathway or their interaction (Figure 9). It is anticipated that this information may provide potential therapeutic molecular targets for the amelioration of peritoneal fibrosis in patients undergoing peritoneal dialysis.

## Methods

### Animal experimental design

Twenty four female mice aged 12 weeks were used. The mice were housed in standard conditions and with free access to food and water. A Peritoneal dialysis mouse model was established according to the report by Aroeira LS *et al.*, as previously described [48]. Briefly, to prevent obstruction and proper drainage of fluid, catheters containing ten holes within 1 cm from the tip were used. Following anesthesia by intra-peritoneal injection of Ketamine (100 mg/kg) and Xylazine (10 mg/kg), the end of catheter was introduced into the peritoneal cavity by the right flank incision, and the port was placed in the subcutaneous space on the dorsum of the mice. One week after the surgery, the mice were divided into 3 groups of 20 mice each. In group one (control group), 1.5 ml of physiologic saline was administered (in this group, 5 mice were sacrificed on day 0 of the experiment). In the second group (PD group), the mice were administered daily 1.5 ml of standard PD fluid containing 4.25% glucose. In the third group (PD + LY group), the mice were administered daily 1.5 ml of standard PD fluid containing 4.25% glucose followed by intra-peritoneal injections of the PI3K/Akt inhibitor, LY294002 (25 mg/kg in 35% DMSO, Sigma). The inhibitor was administered twice a week for 4 weeks, as previously described [49]. All mice were sacrificed at 4 weeks. To avoid injury to the peritoneum of PD mice group, the peritoneal tissues were collected from the upper portion of the parietal peritoneum after sacrificing using 50 mg/kg of pentobarbital sodium anesthesia by intra-peritoneal injection, as described previously [50]. The tissues were then used for various biochemical and morphological studies. The dialysate in the abdominal cavity was also collected and used for various ELISA assays. The animal experiments were approved by the Animal Care and Use Committee of Second Xiangya Hospital of Central South University, Changsha, China.

### Histological and immunofluorescence analysis

For tissue analysis, 4 μm thick paraffin sections from the anterior abdominal wall were taken and processed for Hematoxylin-Eosin (HE) and Masson’s trichrome staining. The thickness of the sub-mesothelial tissue was determined by microscope using a metric ocular; and it was expressed as the mean of 10 independent readings for each mouse section as ref [51]. For confocal imaging analysis, 6 μm thick paraffin sections were pretreated with citrate buffer (10 mM, pH 6.0) for 15 min at 37°C and then incubated with various indicated antibodies at 4°C overnight. Sections were then immuno-stained with FITC-labeled secondary antibodies. The sections were cover-slip mounted and examined with a Zeiss fluorescence microscope.

### Cell culture studies

HMrSV5, a human peritoneal mesothelial cell line (HPMC) [19,20] was a gift from Pierre M Ronco (Tenon Hospital, Paris), and it was maintained in low glucose DMEM medium supplemented with 10% fetal bovine serum (FBS), 100 U/ml penicillin, and 100 mg/ml streptomycin. To study the dose- and time-course effect of TGF-β1 on MMT of HPMCs and the expression of pAkt, Smurf2 and Smad7, 0–10 ng/ml TGF-β1 (R&D Systems) was added at different intervals as indicated (0–72 hrs). To examine the role of Akt in MMT transformation, Smad7 and Smurf2 expression of HPMCs, LY294002 (10–20 μg/ml, Calbiochem) was used to pretreat the cells for 30 min or they were pre-transfected with a dominant negative Akt mutant (K179M) plasmid (Akt-DN, Addgene) for 24 hrs and then the cells were exposed to TGF-β1 (5 ng/ml) for 24–48 hrs.

To investigate the effect of Smurf2 and USP4 on MMT transformation of HPMCs, HPMCs were transfected with Smurf2 siRNA (Ambion), USP4 siRNA (Santa Cruz Biotech), respectively, with or without pWZL Neo Myr Flag AKT1 (Flag Akt plasmid) which is part of a kinase library, and has constitutively active expression (Biovector – Addgene, Plasmid 20422) for 24 hrs. To assess the effect of Akt, Smurf2 and USP4 on TβRI expression, Flag-Akt plasmid, Smurf2 siRNA and USP4 siRNA were used. Cells were transfected with various plasmids or siRNA by following manufacturer’s recommendations where Lipofectamine 2000 Reagent (Invitrogen) was employed. The cells were exposed to TGF-β1 for another 48 hrs. The treated cells were processed for determining their protein expression.

### Immunofluorescence analysis

HPMCs were grown on cover-slips, washed twice with PBS, fixed in 4% paraformaldehyde for 20 min and permeabilized using 0.1% Triton X-100. Anti-ZO-1 (1:100 dilution, Santa Cruz), anti-Vimentin (1:100, Santa Cruz), anti-pAkt (1:80, Cell Signaling) and anti-Smurf2 (1:80, Santa Cruz) antibodies were diluted in a blocking buffer and incubated with cells overnight at 4°C. The cover-slips containing the cells were washed with PBS 3 times, followed by incubation with FITC-labeled secondary antibodies (Santa Cruz) for 2 hrs at 22°C. The cells were then examined using a fluorescence microscope.

### Enzyme-Linked Immunosorbent Assay (ELISA)

Total TGF-β1 protein was determined in PD effluent from the various experimental groups using the Quantikine Immunoassay (R&D Systems, Minneapolis, MN). Fibronectin in the supernatant of cultured HPMCs was measured by the Quantimatrix human fibronectin enzyme-linked immunosorbent assay kit (ELISA) from Chemicon International (Temecula, CA), as described previously [52].

### Real-time Polymerase Chain Reaction (Real-time PCR)

Total RNAs of HPMCs cells and peritoneal tissues were isolated by using Trizol kit (Invitrogen). First-strand cDNAs were generated by two-step RT-PCR (Fermentas Life Science). Real-time PCR was performed using Applied Biosystems 7300 Real-time PCR System and a SYBRgreen PCR reagent kit (Invitrogen). Total RNA was reverse-transcribed and subjected to PCR. The protocol included 94°C for 2 min followed by 40 cycles of the following: 94°C for 15 seconds, 58°C for 30 seconds and 72°C for 30 seconds, and a final extension cycle at 72°C for 10 min. The primer sets used for the various genes are included in Table 1.

**Table 1:** **Real-time PCR primer sequences**

**Primer Name**	**Primer Sequence**
Human Zo-1 (134 bp)	Sense 5′ TGGTGTCCTACCTAATTCAACTCA 3′
	Antisense 5′ CGCCAGCTACAAATATTCCAACA 3′
Mice Zo-1 (97 bp)	Sense 5′ CGAGGCATCATCCCAAATAAGAAC 3′
	Antisense 5′ TCCAGAAGTCTGCCCGATCAC 3′
Human Vimentin (136 bp)	Sense 5′ TTGAACGCAAAGTGGAATC 3′
	Antisense 5′ AGGTCAGGCTTGGAAACA 3′
Mice Vimentin (196 bp)	Sense 5′ ACCGCTTTGCCAACTACAT 3′
	Antisense 5′ TTGTCCCGCTCCACCTC 3′
Human fibronectin (238 bp)	Sense 5′ TACCCTTCCACACCCCAATC 3′
	Antisense 5′ CGGGTATGGTCTTGGCCTAT 3′
Mice fibronectin (184 bp)	Sense 5′ TCTCGGAGCCATTTGTTCCT3′
	Antisense 5′ GAAGCACTCAATGGGGCAAT3′
Human smurf2 (206 bp)	Sense 5′ TAGCCCTGGCAGACCTCTT 3′
	Antisense 5′ CTTGTTGCGTTGTCCTCTGT 3′
Mice smurf2 (114 bp)	Sense 5′ GTGAAGAGCTCGGTCCTTTG 3′
	Antisense 5′ AGAGCCGGGGATCTGTAAAT 3′
Human β-actin (125 bp)	Sense 5′ AGATGTGGATCAGCAAGCAG 3′
	Antisense 5′ GCGCAAGTTAGGTTTTGTCA 3′
Mice β-actin (125 bp)	Sense 5′ AGATGTGGATCAGCAAGCAG 3′
	Antisense 5′ GCGCAAGTTAGGTTTTGTCA 3′

### Nuclear extraction

HPMCs cells were harvested in Phosphate Buffered Saline (PBS), washed twice with cold PBS and then resuspended in 500 μl hypotonic Buffer (20 mM Tris–HCl, pH 7.4, 10 mM NaCl, 3 mM MgCl_2_). They were incubated on ice for 15 min, 50 μl of NP40 added and vortexed for 10 sec. The homogenate was centrifuged at 1,000 × g for 10 min at 4°C. The pellet was designated as the nuclear fraction. The pellet was lysed in 50 μl RIPA buffer (25 mM Tris, pH 7.6, 0.15 M NaCl, 1% NP-40, 1% sodium deoxycholate and 0.1% SDS) containing protease inhibitors. The lysate was centrifuged at 14,000 × g for 30 min, at 4°C. The supernatant was used for Western blot analyses.

### Western blot analysis

Western blot analyses were performed as described previously [7]. Briefly, samples (20 μg protein) were subjected to SDS–PAGE. The proteins were transferred onto nitrocellulose membranes, which were probed with the specific antibodies: anti-ZO-1, anti-vimentin, anti-Smad7 and anti-Smurf2 (Santa Cruz, 1:1000), anti-pan-Akt (phospho T308) antibody (Abcam) also known as anti-pAkt, anti-total Akt, (Cell Signaling, 1:1000) and anti-FN (BD Biosciences, 1:1000). A peroxidase-conjugated goat anti mouse IgG (1: 10 000) was used as a secondary antibody. For detection of other proteins or β-actin, the membranes were treated with Stripping buffer and then re-probed with another antibody or β-actin antibody, the latter served as an internal control.

### Immunoprecipitation/ Western blotting studies with Smurf2 and Smad7

Cell lysates were individually incubated with anti-smurf2 or anti-Smad7 antibody in an IP buffer for 12 hrs at 4°C with gentle orbital rotation. 50 μl of protein A-Sepharose beads were added, and the incubation was extended for another 12 hrs. The beads were washed with the IP buffer and resuspended in SDS loading buffer, and then the entire sample was subjected to 10% SDS-PAGE followed by Western blotting procedures.

### Statistics

Data were expressed as the mean ± SD, and one-way analysis of variance (ANOVA) was carried out. *P* <0.05 was considered statistically significant.
